# Systems pharmacology-based approach to investigate the mechanisms of Danggui-Shaoyao-san prescription for treatment of Alzheimer’s disease

**DOI:** 10.1186/s12906-020-03066-4

**Published:** 2020-09-18

**Authors:** Qihui Wu, Yunbo Chen, Yong Gu, Shuhuan Fang, Weirong Li, Qi Wang, Jiansong Fang, Chuipu Cai

**Affiliations:** 1 Clinical Research Center, Hainan Provincial Hospital of Traditional Chinese Medicine, Guangzhou University of Chinese Medicine, Haikou, 570000 China; 2 Science and Technology Innovation Center, Guangzhou University of Chinese Medicine, Guangzhou, 510000 China; 3 School of Basic Medical Sciences, Guangzhou University of Chinese Medicine, Guangzhou, 510000 China

**Keywords:** Systems pharmacology, Danggui-Shaoyao-san, Alzheimer’s disease, Mechanism of action

## Abstract

**Background:**

Alzheimer’s disease (AD) is the most common cause of dementia in the elderly, characterized by a progressive and irreversible loss of memory and cognitive abilities. Currently, the prevention and treatment of AD still remains a huge challenge. As a traditional Chinese medicine (TCM) prescription, Danggui-Shaoyao-san decoction (DSS) has been demonstrated to be effective for alleviating AD symptoms in animal experiments and clinical applications. However, due to the complex components and biological actions, its underlying molecular mechanism and effective substances are not yet fully elucidated.

**Methods:**

In this study, we firstly systematically reviewed and summarized the molecular effects of DSS against AD based on current literatures of in vivo studies. Furthermore, an integrated systems pharmacology framework was proposed to explore the novel anti-AD mechanisms of DSS and identify the main active components. We further developed a network-based predictive model for identifying the active anti-AD components of DSS by mapping the high-quality AD disease genes into the global drug-target network.

**Results:**

We constructed a global drug-target network of DSS consisting 937 unique compounds and 490 targets by incorporating experimental and computationally predicted drug–target interactions (DTIs). Multi-level systems pharmacology analyses revealed that DSS may regulate multiple biological pathways related to AD pathogenesis, such as the oxidative stress and inflammatory reaction processes. We further conducted a network-based statistical model, drug-likeness analysis, human intestinal absorption (HIA) and blood-brain barrier (BBB) penetration prediction to uncover the key ani-AD ingredients in DSS. Finally, we highlighted 9 key ingredients and validated their synergistic role against AD through a subnetwork.

**Conclusion:**

Overall, this study proposed an integrative systems pharmacology approach to disclose the therapeutic mechanisms of DSS against AD, which also provides novel in silico paradigm for investigating the effective substances of complex TCM prescription.

## Background

Alzheimer’s disease (AD) is a progressive neurodegenerative disease, which starts with mild memory loss and gradually results in severe impairment of broad executive and cognitive functions [1–3]. According to the data from World Health Organization in 2019, approximately 50 million people are suffering from dementia, while AD contributes to 60–70% of these cases [4]. More alarming is a continuous rise in AD cases year by year, leading to increasing disability and high socioeconomic burden. Unfortunately, currently there are only four available anti-AD drugs (Donepezil, Rivastigmine, Galantamine, Memantine) introduced into the market, and no approved disease-modifying treatments (DMTs) exist for AD.

It is worth noting that more and more scientists have turned their attention to the design of multi-targeted drugs instead of the traditional “one target-one drug” perspective, which could intervene the complex AD pathogenesis via multiple molecular targets [5]. As a complementary and alternative medicine, traditional Chinese medicine (TCM), especially the TCM prescription, has been widely applied in Asian countries for the prevention and treatment of complex and degenerative diseases, including AD [6]. Generally, a herbal formula usually comprises multiple components which could act on multiple targets and exert its synergistic effects on disease [7]. In spite of lacking effective options for AD, lots of classical TCM prescriptions (e.g. SuHeXiang Wan, TongLuoJiuNao) have potential as therapeutic drugs for alleviating AD symptoms [8–10]. Danggui-Shaoyao-san (DSS), also known as Toki-shakuyaku-san in Japan and Dangguijakyakan in Korea, is a herb combination employed to improve cognitive function, which consists of six Chinese herbs including *Paeoniae Radix Alba (BaiShao, BS), Angelica Sinensis Radix (DangGui, DG), Atractylodis Macrocephalae Rhizoma (BaiZhu, BZ), Chuanxiong Rhizoma (ChuanXiong, CX), Alismatis Rhizoma (ZeXie, ZX), and Poria (FuLing, FL)*. According to a meta-analysis conducted on randomized controlled trials (RCTs), DSS has a positive effect on the scores of Mini-Mental State Examination (MMSE) and activities of daily living (ADL) of AD patients [11]. Clinical study found that the regional cerebral blood flow (rCBF) of AD patients in the posterior cingulate were significantly higher and orientation to place tended to improve after treatment with DSS [12]. Besides, previous pharmacological studies have deciphered that DSS could exert potentially therapeutic effects for AD via multiple mechanisms such as disrupting the aggregation of Aβ, attenuating inflammatory reaction, and adjusting the mitochondrial membrane permeability **(**Table 1**)**. For instance, a study in 2014 has reported that DSS could improve learning and memory capacity in female SAMP8 mice through modulating estrogen, nitric oxide, and glycine in plasma or hippocampal tissue [13]. Furthermore, Kou et al. demonstrated that DSS ameliorated memory dysfunction and protected the ultrastructure of cortex changed by aging, which may be beneficial for the treatment of AD [14]. Despite current reported therapeutic mechanisms, the underlying mechanism of actions (MOAs) and active components of DSS against AD still remain indistinct. Thus, it is necessary to comprehensively investigate the molecular mechanism of DSS for treatment of AD and deeply understand their synergistic effects on multiple pathways.

**Table 1:** Current in vivo studies on the therapeutic mechanism of actions of DSS against AD

Year	Brief description	PMID
2020	Ameliorating cognition deficits in APP/PS1 mice via increasing DHA content and regulating oxidative stress and inflammation	32084555
2014	Improving learning and memory in female SAMP8 mice via modulating estrogen, nitric oxide, and glycine in plasma or hippocampal tissue	24757492
2011	Improving cognition of the rats which might be related to attenuate inflammatory reaction and reduce cell apoptosis in the hippocampus	22375398
2010	Improving spatial learning and memory deficits in mice, reverse the inhibition of Long-term potentiation in hippocampal slices, prevent aggregation of Aβ, and even disrupt the aggregated Aβ fibrils	20117199
2008	Upregulating Bcl-2 level and downregulate Bax level which might in turn adjust the mitochondrial membrane permeability, attenuate cytochrome c and its release into cytosol, following the suppression of caspase activation	18093848
2005	Ameliorating memory dysfunction, modulate metabolism of monoamine neurotransmitters and protect the ultrastructure of cortex changed by aging	15707771
2005	Reducing the Abeta25–35-induced neuronal death and the lipid peroxidation which has a protective effect against Abeta25–35-induced neuronal damage	16106382

Systems pharmacology is an emerging discipline which combines in silico network-based tools and experimental assays, aiming to elucidate the changes in the functions and reactions of human body induced by medicines [15]. Based on the holistic principle, the herbal formulas in TCM show the characteristics of multi-component and multi-target in therapy in contrast to synthetic drugs. Due to the complexity of ingredients and targets, conventional experimental approaches are time-consuming and expensive for TCM research. Systems pharmacology offers effective strategy to explore the multi-component network target research pattern of Chinese herbal medicine and formula [16–18].

**Fig. 1:**
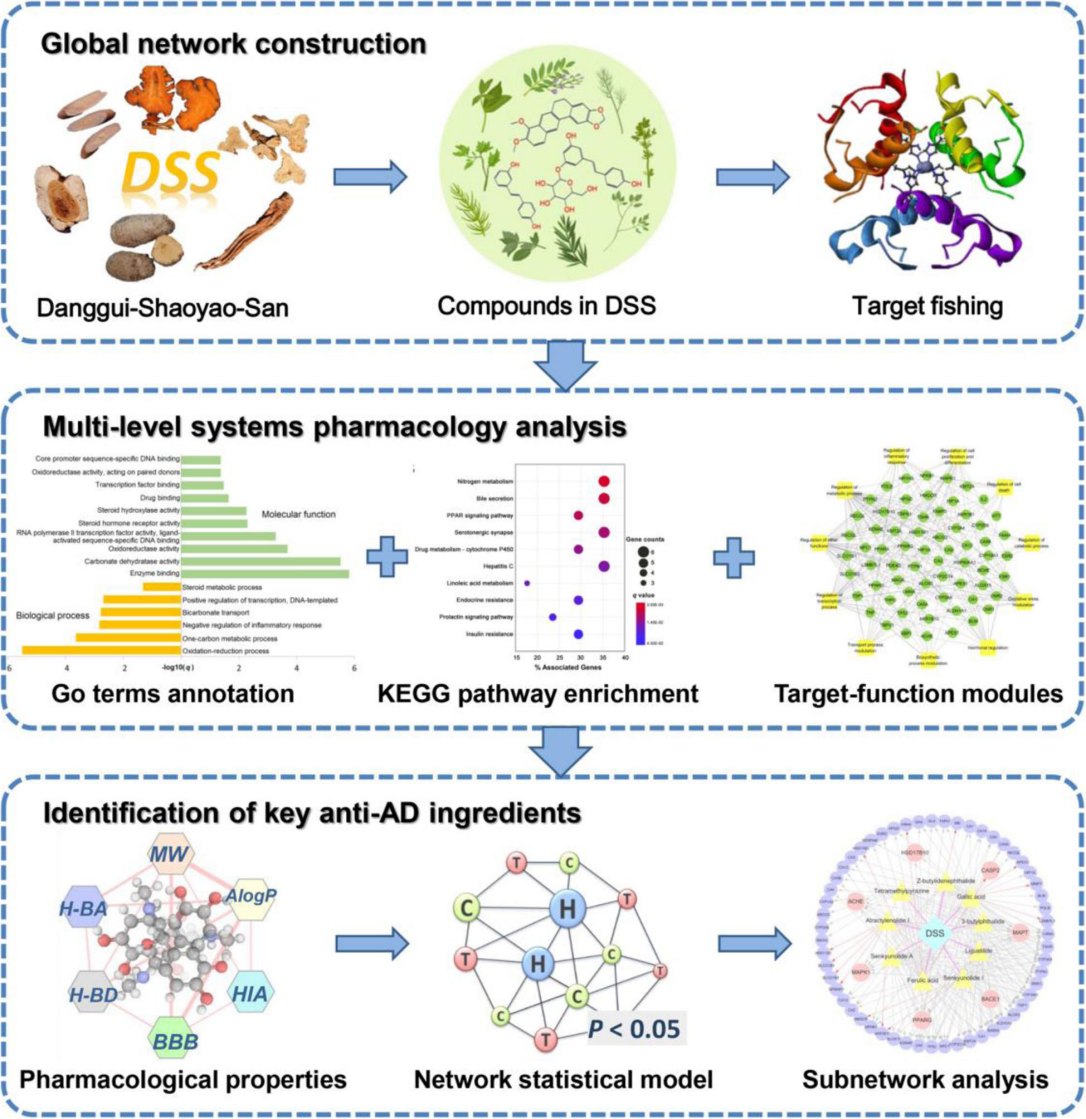
Schematic diagram of the systems pharmacology approach for deciphering the pharmacological mechanisms of DSS for treatment of AD

In this study, we attempted to systematically explore the MOAs of DSS for treatment of AD through an integrated systems pharmacology framework **(**Fig. 1**)**. Firstly, we collected the chemical ingredients of DSS with known protein targets from our previous integrated natural products database [19]. We further computationally predicted the putative targets of DSS via a network-based inference method. Subsequently, a global drug-target network of DSS was constructed by incorporating the known and predicted DTIs. Furthermore, multi-level systems pharmacology analysis methods, including molecular-function analysis, biological process analysis, target-function modules analysis, and KEGG pathway enrichment were performed to elucidate the MOAs of DSS against AD. Additionally, we developed a network-based model for identifying the active anti-AD components of DSS by mapping the high-quality AD disease genes into the global drug-target network. After integrating high-performance liquid chromatography (HPLC) analysis data, drug-likeness analysis, human intestinal absorption (HIA) and blood-brain barrier (BBB) penetration assessment, and network statistical model prediction results, we highlighted the key anti-AD ingredients in DSS and illustrated the specific synergistic mechanisms through subnetwork analysis.

## Methods

### Manual curation of genes associated with AD

The genes associated with AD were collected from six authoritative databases: 1) the Malacards database (https://www.malacards.org); 2) the Comparative Toxicogenomics Database (CTD) [20]; 3) DisGeNET [21]; 4) the GWAS Catalogue [22]; 5) the Human Gene Mutation Database (HGMD) database [23]; and 6) AlzBase database (http://alz.big.ac.cn/alzBase/summary/Gene). Only genes labeled with “Alzheimer’s disease” were extracted from the databases mentioned above. Additionally, for the AlzBase database, the top 100 genes were preserved for further investigation. Finally, 299 AD-related genes (Supplementary Table S1) were integrated after removing the duplicates.

### Collection of herbal ingredients and known protein targets

The ingredients and their protein targets in each herb of DSS were collected from our previous integrated database [24], which includes experimental validated DTIs of natural products extracting from over 2000 literatures and five authoritative compound-protein interaction databases: ChEMBL (v21) [25], BindingDB [26] (accessed in Sep. 2017), STITCH [27], the Herbal Ingredients’ Target Database (HIT) [28], and the Traditional Chinese Medicine Integrated Database (TCMID) [29]. Consequently, a total of 1042 herbal ingredients in DSS were obtained. The corresponding number of the collected compounds in DG, CX, FL, ZX, BS, BZ is 549, 351, 84, 42, 125, 170, respectively. The detailed structural information of the 1042 herbal ingredients is provided in Supplementary Table S2.

### Network-based target prediction for DSS

In this study, target fishing is carried out to predict targets based on known DTIs via a balanced substructure-drug-target network-based inference (bSDTNBI) method [30]. We previously developed predictive network models to identify new targets of natural products with bSDTNBI method, which prioritizes potential targets utilizing resource-diffusion processes for both known and new natural products [31]. During the process, four parameters (α = β = 0.1, γ = − 0.5, and k = 2) of bSDTNBI were adopted. The first parameter α was introduced to balance the initial resource allocation of different node types, while β was utilized to adjust weighted values of different edge types. The third parameter γ was applied to balance the effect of hub nodes in resource diffusion processes, and the last parameter κ represented the number of resource-diffusion processes. We subsequently calculated four types of molecular fingerprints for each compound based on PaDEL-Descriptor (version 2.18) [32], including MACCS, PubChem, Substructure (FP4) and Klekota-Roth (KR). Compared with the other three generated predictive models, bSDTNBI_KR performed best given its highest values of the area under the receiver operating characteristic curve (AUC = 0.959). Eventually, the bSDTNBI_KR predictive model was chosen to identify new targets of each natural product. In this study, the top 20 putative targets for each compound with known targets were selected (Supplementary Table S3).

### Identification of key anti-AD ingredients in DSS

The key anti-AD ingredients in DSS were determined by four steps. Firstly, we obtained the main components in DSS according to the HPLC analysis results reported in previous references [33, 34]. Subsequently, we applied the Lipinski’s Rule of Five (molecular weight < 500, AlogP < 5, H-bond donor < 5, H-bond acceptor < 10) [35] to assess the drug-likeness of these components. Thirdly, a machine learning-based ADMET Simulator tool [36] was further utilized to evaluate their BBB penetration and HIA properties. The 166-bit fingerprint descriptor MACCS was used for chemical structure representation. Finally, we built a network-based statistical model to prioritize anti-AD compounds in DSS based on drug–target network and AD-related genes. We hypothesize that a compound would exert high potential for the treatment of AD if its targets tend to be encoded by AD-related genes [15]. Fisher’s exact test was applied to calculate the statistical significance of the enrichment of AD-related proteins in target profiles of each ingredient in DSS. The *P*-values were corrected by Benjamin–Hochberg method [37]. Predicted compound–AD pairs with adjust-P (*q*) value lower than 0.05 were regarded as significant.

### Network construction and enrichment analysis

The drug-target (D-T) network and target-function (T-F) network was constructed to explore the molecular mechanism of DSS for treatment of AD. Networks were generated and analyzed by Cytoscape (v3.2.1, http://www.cytoscape.org/) and Gephi (v0.9.2, https://gephi.org/). In each graphical network, compounds or genes or functional modules were presented by nodes, while interactions were encoded by edges. The degree of each node was calculated, which represents the number of edges linked to it, characterizing the most important nodes in a network. Gene ontology (GO) term and KEGG pathway enrichment analysis were performed by DAVID (The Database for Annotation, Visualization and Integrated Discovery, http://david.abcc.ncifcrf.gov) and Omicshare webservers. Significantly enriched pathways in drug-target set comparing to the genome background were defined by hypergeometric test. The calculated *p*-value (*q*) was gone through FDR Correction and *q* < 0.05 was considered as statistically significant. The statistical analysis was performed by Python platform (v3.2, http://www.python.org/).

## Results

### Overlap analysis of herbal ingredients and targets in DSS

**Fig. 2:**
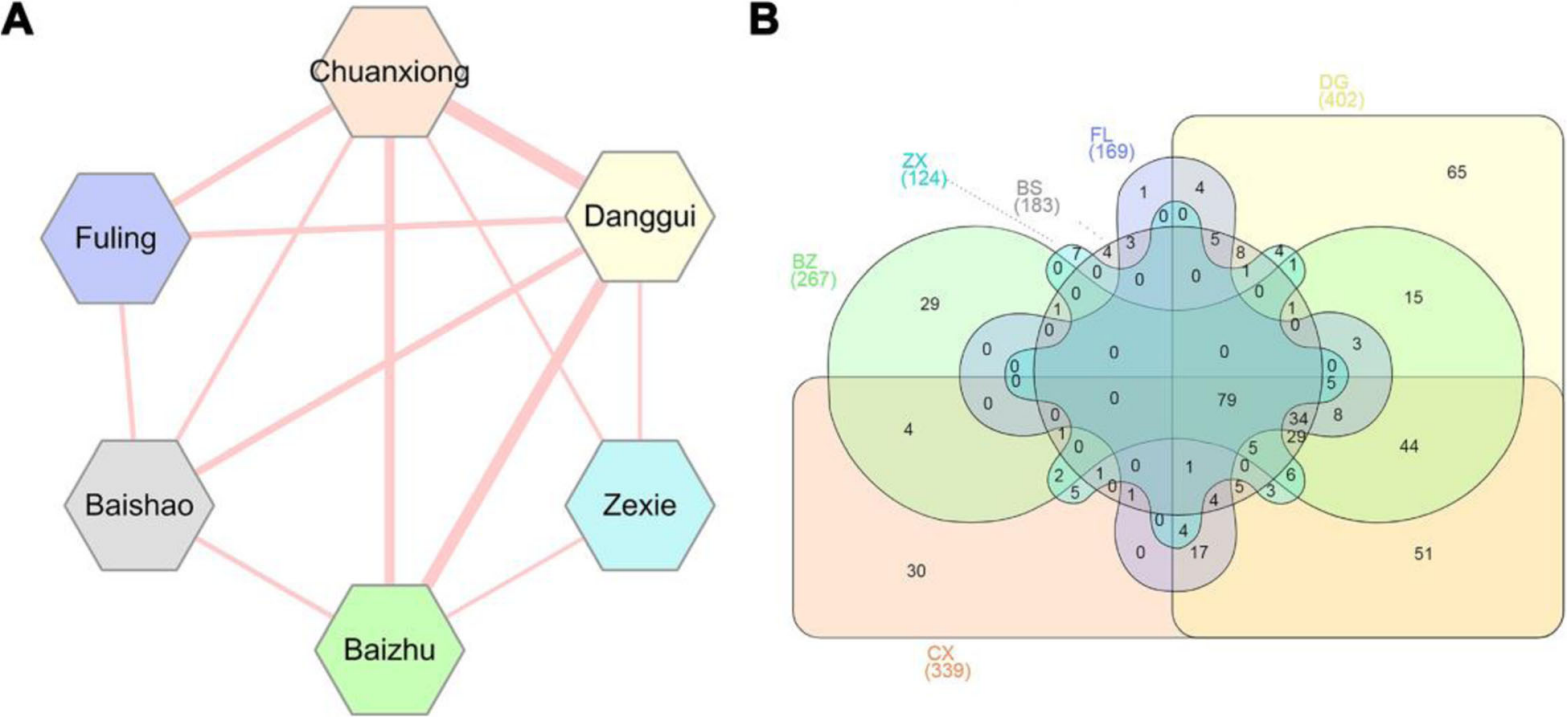
Overlap analyses of herbal ingredients (**a**) and corresponding targets (**b**) among the six herbs in DSS prescription. The thickness of connected lines between two herbs is proportional to overlapped ingredients. The corresponding number of ingredients for each herb is 547 (Danggui, DG), 170 (Baizhu, BZ), 83 (Fuling, FL), 42 (Zexie, ZX), 118 (Baishao, BS) and 349 (Chuanxiong, CX), while the corresponding number of targets for each herb is 339(CX), 402(DG), 183(BS), 267(BZ), 169(FL) and 124(ZX), respectively

“Jun-Chen-Zuo-Shi”, also known as the “sovereign-minister-assistant-courier” principle [38], is a key theory of TCM which guides physicians to formulate herbal medicine [39]. This principle represents the different roles played by each herb in a formula as well as its change rules on effect after compatibility of medicines. In the DSS prescription, BS, DG and CX belong to Jun-Chen class (primary role), while FL, ZX and BZ serve as the Zuo-Shi class (subsidiary role). To figure out the specific formulate principle of DSS for treatment of AD at a molecular level, overlap analyses of herbal ingredients and their targets were performed. Figure 2a indicates the relationship of the six herbs based on the number of overlapped ingredients between each two herbs. Obviously, multiple herbs share large common compounds. Among the six herbs in DSS, DG and CX own the highest number of duplicate compounds (*n* = 48), followed by DG and BZ (*n* = 27), CX and BZ (*n* = 19). Meanwhile, Fig. 2b shows these six herbs in DSS covered 491 targets in total. Among them, DG has the greatest number of targets. Interestingly, we found that 79 common targets existed in all of these six herbs simultaneously, which implied DSS might magnify its therapeutic effect via modulating these common targets. These targets include estrogen receptor, peroxisome proliferator-activated receptor (PPAR), inflammatory factor, transcription factor, cytochrome P450 family and lipoxygenases. Taking PPARA as an example, a recent study has demonstrated that PPARA is an important factor regulating autophagy in the clearance of Aβ. Activation of PPARA-mediated autophagy could reduce AD-like pathology and cognitive decline in a murine model [40]. The detailed information of the common targets can be found in Supplementary Table S4.

### Construction of drug-target network for DSS

To develop a global drug-target network for DSS, all the chemical ingredients in DSS were mapped into a known DTI network of natural products to acquire experimental validated DTIs from our previous integrated database [19]. Moreover, the predicted DTIs were obtained with the bSDTNBI method. Finally, the network of DSS contains 19,293 DTIs connecting 937 unique compounds with 490 targets (37 AD targets and 453 non-AD targets) (Supplementary Table S5).

**Fig. 3:**
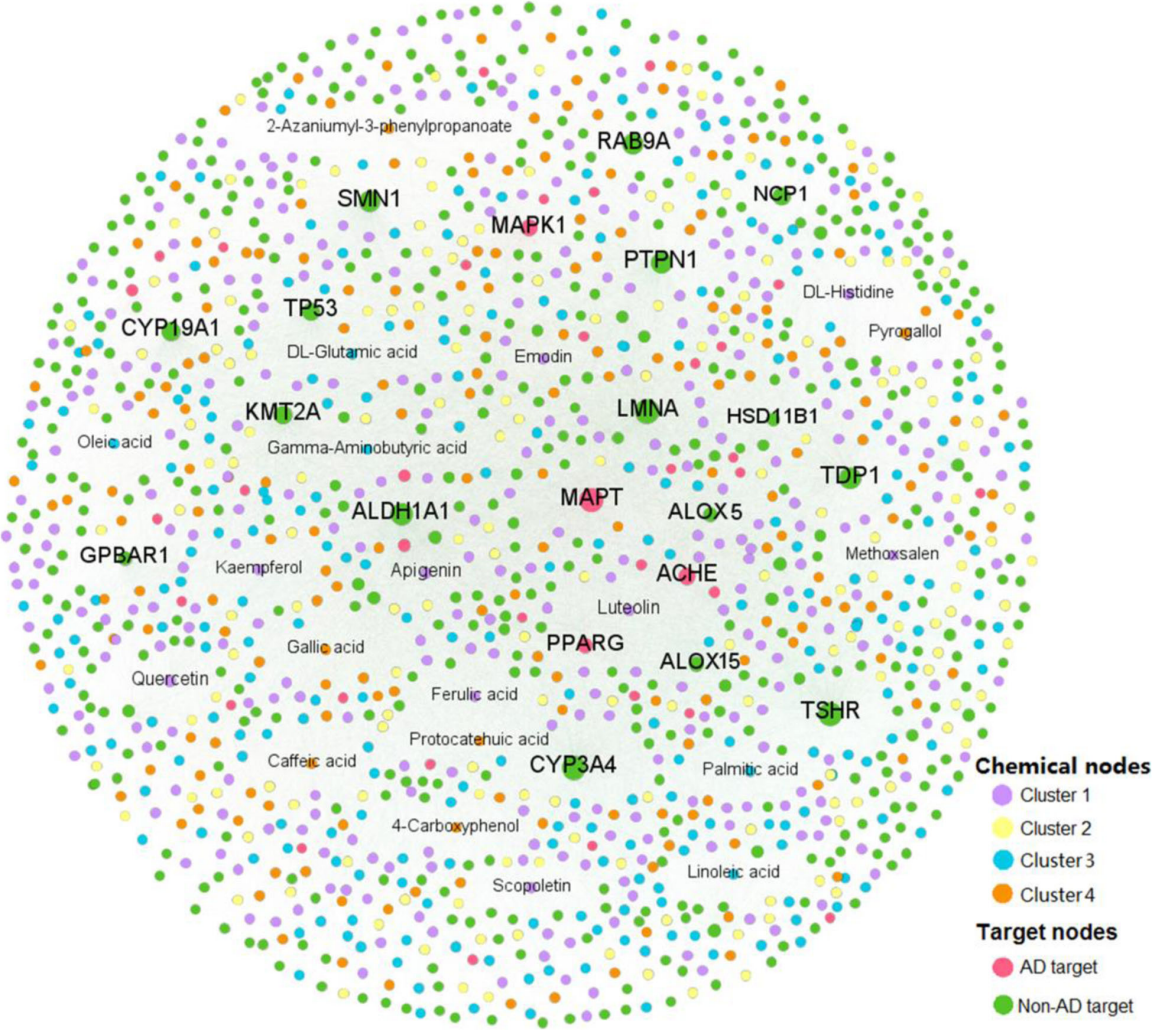
Bipartite drug-target interaction (DTI) network of DSS consisting of 19,293 DTIs. The label font size and node size are proportional to degree. Labels of the top 20 compounds and targets with highest degrees are displayed. The compounds grouped into different chemical scaffold clusters are displayed in different colors (Supplementary Table S5)

As illustrated in Fig. 3, most compounds are connected to multiple targets. The average target degree (*D*) for a compound is 20.6, while the average drug degree (*K*) of a target is 39.4. Among the 937 compounds, the top 10 with largest target degree (*D*) are quercetin (CID5280343, *D* = 100), apigenin (CID5280443, *D* = 70), luteolin (CID5280445, *D* = 59), kaempferol (CID5280863, *D* = 47), caffeic acid (CID689043, *D* = 43), gallic acid (CID370, *D* = 40), DL-glutamic acid (CID611, *D* = 37), Gamma-aminobutyric acid (CID119, *D* = 36), emodin (CID3220, *D* = 35) and scopoletin (CID5280460, *D* = 32). Within these compounds, 4 out of them have AD-labeled target degree greater than 5, including quercetin, apigenin, luteolin and Gamma-aminobutyric acid. Indeed, evidence is emerging that these compounds are highly correlated with AD treatment. For example, quercetin has been reported to reverse histological hallmarks of AD and exert a protective effect on cognitive and emotional function in aged 3xTg-AD mice [41]. Moreover, apigenin owns potent neuroprotective properties to protect iPSC-derived AD neurons via reducing the frequency of spontaneous Ca2^+^ signals and significantly reducing caspase-3/7 mediated apoptosis [42]. Meanwhile, 16 proteins are targeted by more than 300 compounds (*K* > 300). Among them, MAPT (*K* = 905), ACHE (*K* = 448), MAPK1 (*K* = 435) and PPARG (*K* = 320) are AD-related targets, which have been confirmed to play a vital role in AD pathobiology. For instance, a previous research has indicated that MAPK1 (ERK2) is positioned to phosphorylate normal tau which could be involved in the generation of paired helical filaments in AD [43]. Besides, arachidonate 15-lipoxygenase (ALOX15), a protein targeted by 276 compounds (*K* = 276) across all the 6 herbs in DSS, is an important enzyme to regulate the homeostasis of DHA metabolism and esterification that are associated with cognitive function [44]. A recent study found that the mean densities of ALOX15 were improved by DSS treatment in both hippocampus and cortex of APP/PS1 mice [45]. These findings suggest the potential therapeutic targets of DSS for exerting anti-AD effects.

### Target-function network (T-F network)

**Fig. 4:**
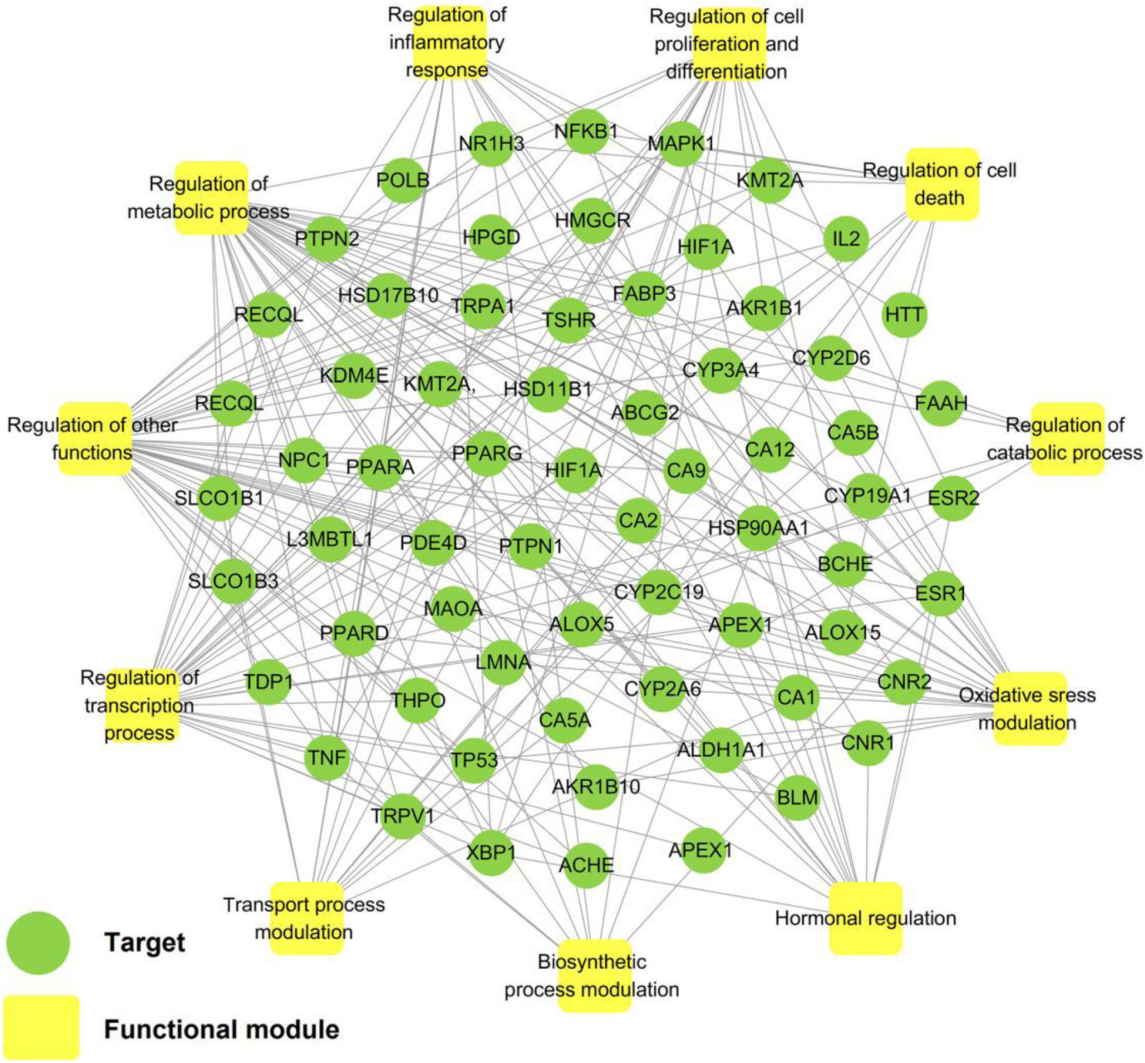
Target-function network (T-F network) of DSS. A target protein and a functional module will be linked if the target appears in that biological process

Different network regions may underlie different biological pathways, processes or cellular localizations. Here we extracted the targets of DSS with degree ≥40 to construct a target-function (T-F) network. The T-F network demonstrates AD-related biological processes and associated targets based on the functional annotation bioinformatics microarray (DAVID 6.8) analysis [46]. As depicted in Fig. 4, this network consists of 209 target-function pairs linking 66 targets with 11 AD-related functional modules. The average number of functional modules for each target has reached up to 6, and 34 targets are associated with more than 4 functional modules. These modules include oxidative stress, metabolic process, inflammatory response, transport process, transcription process, hormonal regulation, biosynthetic process, catabolic process, cell proliferation and differentiation, and cell death, indicating the potential AD-related pathological process mediating by DSS. For instance, the T-F network suggests that DSS may regulate oxidative stress via reducing oxidation and regulation of nitric oxide. AD is highly correlated with oxidative stress and increased production of reactive oxygen species (ROS) has direct effect on synaptic activity and neurotransmission in neurons, hence leading to cognitive dysfunction [47]. Previous in vivo study had confirmed that DSS could significantly relieve oxidative stress, such as reducing the Aβ_25–35_-induced lipid peroxidation and possessing protective effect against neuronal damage [14].

### GO term and KEGG pathway enrichment analysis

**Fig. 5:**
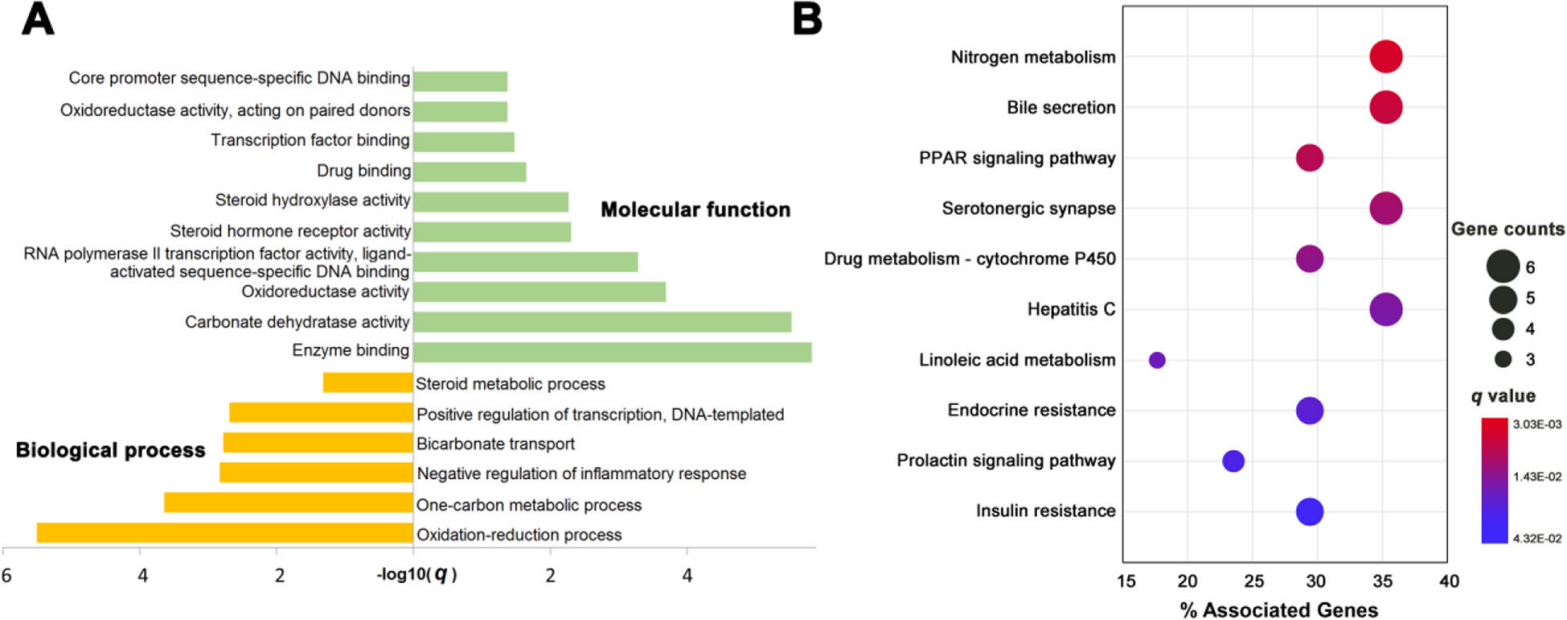
GO terms annotation (**a**) and KEGG pathway enrichment (**b**) of the targets (K ≥ 40) in the drug-target network of DSS

In this study, GO term enrichment analysis was performed on the 73 targets with degree equal or larger than 40. Figure 5a shows the significantly enriched terms (*q* < 0.05) in biological process (BP) and molecular function (MF) categories. Obviously, most of the biological processes are related with AD, such as the negative regulation of inflammatory response (BP, GO:0050728) [48] and oxidation reduction process (BP, GO:0055114) [49]. Previous literature has revealed that anti-inflammatory drugs could significantly reduce the incidence of AD [48]. Moreover, accumulating evidence demonstrates that anti-oxidations are potentially promising strategies for safe and efficient treatment of AD [49]. Similarly, it is worthy to note that a great number of targets are associated with a variety of MF terms, which plays a role in the pathogenesis of AD. For example, carbonate dehydratase (MF, GO:0004089), has been indicated to serve as a novel therapy for AD [50]. In addition, transcription factor (MF, GO:0008134) such as XBP1s, was also reported to restore hippocampal synaptic plasticity and memory by control of the Kalirin-7 pathway in Alzheimer model which may be therapeutic implications in AD pathology [51]. The detailed information of GO terms analysis data were provided in Supplementary Table S6.

Generally speaking, drug action does not only have regulatory effects on their related targets, but also play a role for metabolic enzymes and downstream proteins, as well as some pathways that are related with the specific disease [52]. To identify the significant pathways involved in DSS for treatment of AD, targets (*K* ≥ 40) in the drug-target network were mapped onto their related pathways based on KEGG annotation (Supplementary Table S7). Figure 5b showcases the top 10 significant KEGG pathways (*q* < 0.05), and some of them are intensively correlated with AD treatment. For instance, PPAR signaling pathway (ko03320) has been demonstrated to increase phosphorylation of fibroblast growth factor 14 in the Tg2576 mouse model of AD [53]. Furthermore, a recent study shows that bile secretion (ko04976) is associated with “A/T/N”(amyloid, tau, and neurodegeneration) AD biomarkers, providing further support for a role of bile acid pathway in AD pathophysiology [54]. Taken together, these results indicate the novel pathways and biological processes that may be regulated by DSS for exerting anti-AD effects, which is worthy of further research by in vivo and in vitro experiments.

### Integrated pathway analysis of DSS related to AD pathogenesis

**Fig. 6:**
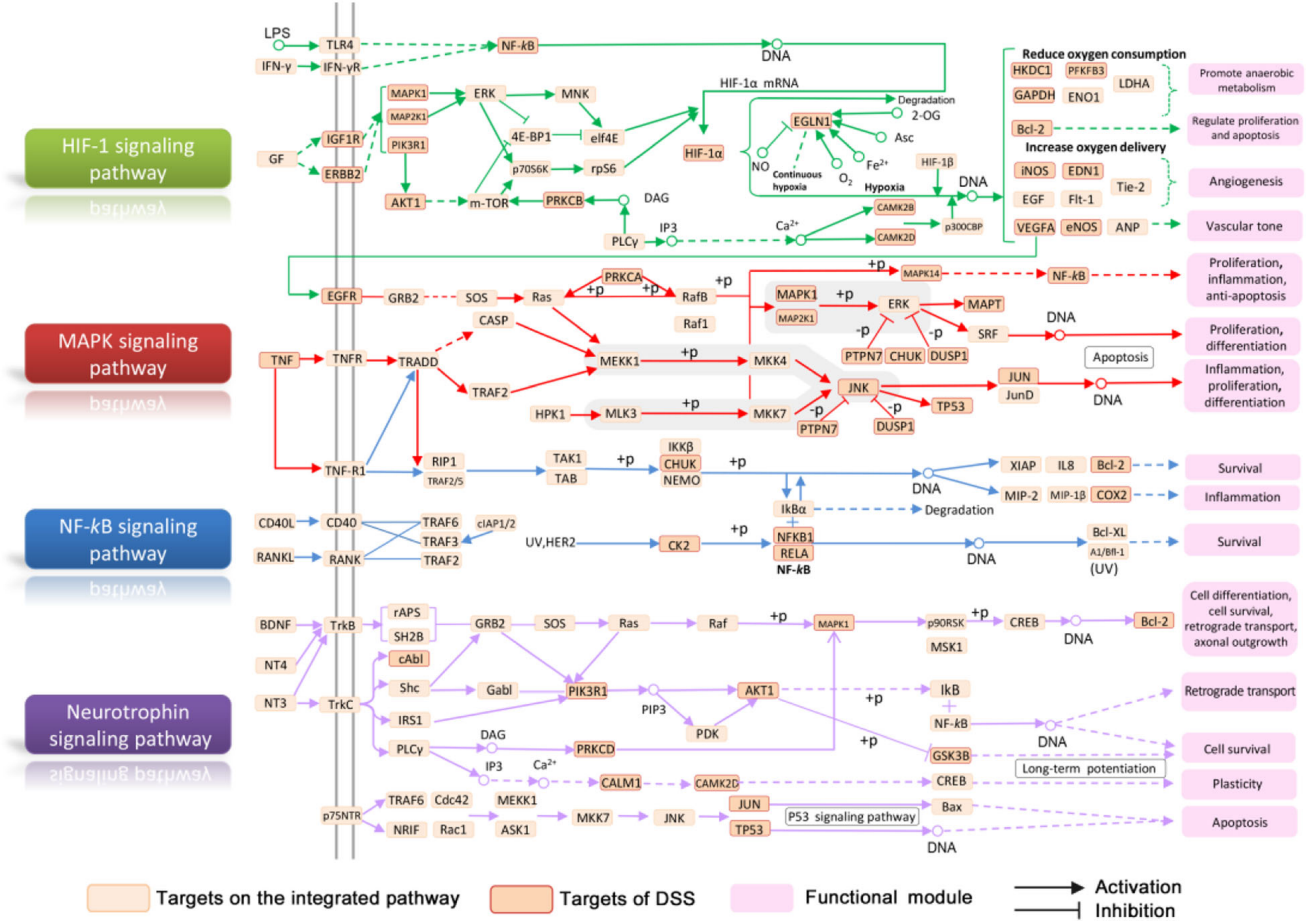
AD-integrated pathway and functional modules. The light orange nodes represent targets on the integrated pathway while the dark orange nodes indicate the protein targets of DSS formula. The pink nodes represent the functional modules

In this analysis, protein targets of DSS were mapped into the pathways that directly related to the pathological process of AD to construct an AD-integrated pathway **(**Supplementary Table S8**)**. Several functional modules are involved in the integrated pathway, such as inflammation, oxidative stress, apoptosis, and neurotrophin modulation. Here, four representative modules (Fig. 6) were chosen to illustrate the potential therapeutic mechanisms of DSS on the metabolic pathways towards AD.

#### Anti-oxidative stress module

Oxidative stress has been considered as a contributing factor in the pathogenesis and progression of AD. The increased ROS directly affects synaptic activity and neurotransmission in neurons leading to cognitive dysfunction [47]. There is increasing evidence that hypoxia-inducible factor 1 (HIF-1) possesses neuroprotection in the setting of neuronal insults. HIF-1 promotes glycolysis and glucose metabolism, thus helping to produce reductive equivalents of NADH/NADPH which counters oxidative stress [55]. Figure 6 shows that DSS prescription could act on multiple targets on HIF-1 signal pathway (e.g. HIF-1α, IGF1R, ERBB2), indicating its potential pathway of oxidative stress regulation for AD treatment and prevention.

#### Neuroinflammation regulation module

Neuroinflammation is an important process in neurodegeneration in AD, involving in a vicious cycle of amyloid deposition, neuronal damage, tangle formation, and death [56]. Evidences indicate that Aβ-induced neuroinflammation is associated with high levels of pro-inflammatory cytokines, including TNF-α, interleukin-6 (IL-6), and interleukin-1β (IL-1β) [57]. P38 mitogen-activated protein kinase (MAPK) is a crucial target for chronic inflammatory diseases [58]. Previous studies imply that MAPK is significantly involved in glial activation and subsequent neuroinflammation, resulting in chronic neurotoxicity [58]. As exhibited in Fig. 6, compounds in DSS target to several key proteins in the MAPK signaling pathway, suggesting the important pathway that DSS may participate for the regulation of neuroinflammation and amyloid genesis.

#### Modulation functions related to NF-κB signaling

Nuclear factor-kappa B (NF-κB) plays pivotal role in gene regulation and implicates in oxidative stress, apoptosis, and inflammation [59]. NF-κB can be activated in most cell types, including neurons, astrocytes, microglia, oligodendrocytes and endothelial cells of neurovascular and cerebrovascular units. Recent researches demonstrated that NF-ĸB signaling pathway plays key physiological role in central nervous system, which serves important functions in cellular responses to neuronal injury and synaptic plasticity [60]. Figure 6 illustrated that lots of compounds in DSS act on the targets of NF-ĸB signaling pathway, indicating the potential AD therapeutic mechanism mediated by DSS. Recent in vitro study showed that DSS has a protective effect on neuroinflammation in lipopolysaccharide (LPS)-stimulated BV-2 microglia cells through the TLRs/NF-κB signaling pathway [61].

#### Neurotrophin modulation module

Neurotrophic factors, such as brain-derived neurotrophic factor (BDNF) and neurotrophin 3 (NT3) are critical to the maintenance of the nervous system in the adult brain [62]. Growing evidences suggested that up-regulation of BDNF can relieve cognitive impairments and learning deficits in AD [63], while NT3 can promote the proliferation and differentiation of bone marrow-derived NSCs into cholinergic neurons and elevate the levels of acetylcholine (ACh) [64]. Figure 6 shows that protein targets of DSS are enriched in the neurotrophin signal pathway, which suggests the critical role that DSS may play on neurotrophin modulation.

### Prescription simplification: uncovering the key anti-AD ingredients in DSS

The complexity of ingredients as well as their intricate target network in TCM prescription is always a huge challenge that hinders the translation from pre-clinical testing results to clinical outcome. Although previous HPLC analysis had preliminary estimated the main active components in DSS [33, 34], the specific ingredients that exert therapeutic effect against AD are difficult to determine. In this section, we further narrow up the study scope of compounds in DSS, for identifying the key anti-AD ingredients and optimum drug combinations. After the four-step screening (see Methods), nine key anti-AD ingredients in DSS were finally highlighted, including 3-butylphthalide, ligustilide, senkyunolide I, Z-butylidenephthalide, senkyunolide A, atractylenolide I, tetramethylpyrazine, ferulic acid, and gallic acid (Table 2). According to the previous reported HPLC analysis results, all of the nine ingredients are the major constituents in DSS extract (Table 2) [33, 34]. To investigate the basis of compositions against AD, we searched the previous literature for these 9 ingredients and surprisingly found that 6 of them were confirmed to possess anti-AD effects (Table 2). For instance, tetramethylpyrazine (TMP), one of the major bioactive compounds purified from CX, shows great potential against AD, which could restore the function of cholinergic neurons and protect against memory loss in AD rat model [65]. Besides, ligustilide was reported to have neuroprotective effect via inducting α-Secretase processing of both APP and Klotho in AD mouse [66]. The high hit rate of our prediction suggests the AD therapy potential of the rest three unreported ingredients (atractylenolide I, senkyunolide I, senkyunolide A), which deserve to be validated by experimental assays in the future. Interestingly, we also found that these 9 components are mainly derived from three herbs (DG, CX, BS) in DSS, which serve as “JUN” (BS) and “CHEN” (DG, CX) role, indicating the rationality of DSS in formulation.

**Table 2:** Nine key anti-AD ingredients in DSS after screening by drug-likeness analysis, human intestinal absorption and blood brain barrier assessment, and network statistical model prediction

Compound	Pubchem ID	HIA	BBB	MW	AlogP	H-BA	H-BD	*q* value	Structure	Herb	Contents	PMID
Ferulic acid	445858	0.96	0.60	194.19	1.5	3	2	6.76E-03		DG; CX; FL	0.25	29896095
Atractylenolide I	5321018	0.99	0.97	230.31	3.51	2	0	9.72E-03		BZ	0.04	N/A
Ligustilide	5319022	1	0.99	190.24	2.87	2	0	6.76E-03		DG; CX	2.54	29163135
Tetramethylpyrazine	14296	0.99	1	136.2	1.71	2	0	9.72E-04		CX	N/A	28633346
Senkyunolide I	11521428	0.94	0.81	224.26	1.04	4	2	6.76E-03		CX	0.9	N/A
Senkyunolide A	3085257	1	0.99	192.26	2.75	2	0	3.68E-02		DG; CX	2.14	N/A
Gallic acid	370	0.78	0.53	170.12	0.5	4	4	1.36E-04		CX; BS; FL	0.48	30447302
3-butylphthalide	61361	1	0.99	190.24	3.09	2	0	6.76E-03		DG; CX	0.35	28587340
Z-butylidenephthalide	642376	1	0.99	188.23	3	2	0	6.76E-03		DG; CX	0.16	25735452

Note: the *q* values indicate the significance between compounds and AD calculated by the network statistical model; the values of HIA (Human Intestinal Absorption) and BBB (Blood Brain Barrier) refer to the positive probability predicted by ADMET Simulator [36]; the contents refer to contents (mg/g) of each compound in DSS extract determined by HPLC analysis [34]; the PMID links to the published anti-AD literature of each compound; MW: Molecular Weight; H-BA: H-Bond Acceptor; H-BD: H-Bond Donor

**Fig. 7:**
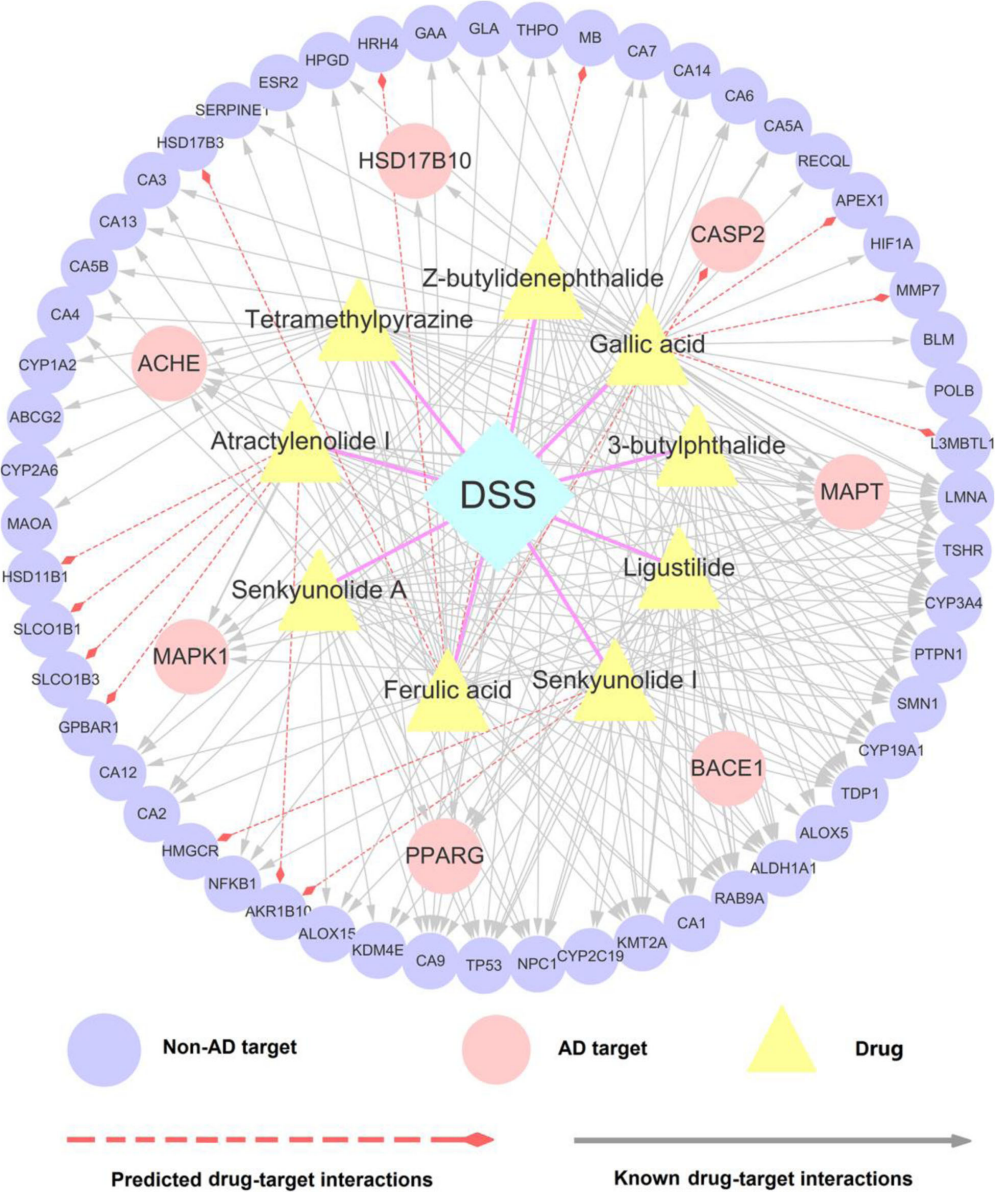
A drug-target subnetwork of the 9 key anti-AD ingredients in DSS connecting to 62 target proteins

To explore the MOAs for the nine key ingredients toward AD, a subnetwork was constructed by extracting the DTIs of these compounds from the global drug-target network of DSS. As illustrated in Fig. 7, the specific D-T subnetwork is composed of 194 known DTIs and 14 predicted DTIs connecting 9 compounds with 62 target proteins. The 9 ingredients are connected to multiple targets with the average degree of 3.4. Among them, gallic acid (CID370) has the highest number of target connections, binding with 37 known targets and 3 predicted targets, which indicate its potential multi-target anti-AD mechanism. For example, MMP7, one of the three predicted targets, has been demonstrated to play an important role in the defensive mechanism against the aggregation of Aβ_1–42_, giving rise to the pathology of AD [67]. Remarkably, the 9 ingredients in DSS share multiple overlapped protein targets. Five proteins, including TSHR, TOP1, CYP3A4, LMNA and MAPT, are simultaneously targeted by all the 9 ingredients, suggesting that DSS may mainly exert its anti-AD effect through the synergistic effect of these components. Taking MAPT as an example, published studies have revealed that MAPT gene polymorphisms could increase the AD risk, which is highly related with the deposition of Aβ proteins [68–70].

Overall, the prescription simplification strategy applied here provides a new perspective for uncovering the pivotal constituents in DSS and their specific molecular mechanism for treatment of AD.

## Discussion

To date, the prevention and treatment of AD remains a huge challenge. In last two decades, although more than 400 clinical trials have been conducted for AD treatment, over 99% failed in clinical trials owing to the adverse events and limited benefits [71]. Therefore, it is urgent to find a new and effective therapy for AD from a broader perspective. TCM prescriptions emphasize the rebalancing of interactions among various disease-related factors within the abnormal body. As one of the representative prescriptions, DSS has been demonstrated to be effective for improving cognitive function of AD patients [11]. However, since TCM prescriptions contain complex chemical components interacted with multiple targets, the specific pharmacological mechanisms of DSS against AD are still difficult to illustrate.

In this study, we developed an integrative systems pharmacology framework to decipher the MOAs of DSS against AD. We first performed an overlapped analysis among the herbal ingredients as well as potential targets and constructed a global drug-target network of DSS. We further explored the potential MOAs of DSS for AD treatment through multiple enrichment analyses, including target-function modules analysis, GO terms annotation, KEGG pathway enrichment, and AD-integrated pathway. Finally, we proposed a prescription simplification strategy, which integrates HPLC analysis results, drug-likeness analysis, pharmacological properties prediction, and network-based statistical model, to uncover the key anti-AD compounds in DSS. We highlighted 9 key ingredients in DSS that play synergistic role against AD in the subnetwork.

Systems pharmacology analysis suggests that DSS participates in the regulation of several important biological pathways related to AD pathogenesis, such as the oxidative stress, inflammatory reaction processes and neurotrophin signaling pathway (Fig. 4 and 5a). Recently, Huang et al. [45] found that DSS extract prominently lessened the abnormal activity of ROS and malondialdehyde (MDA) level, and increased the intracellular superoxide dismutase (SOD) and glutathione (GSH) level in APP/PS1 mice. Moreover, following treatment with DSS, the levels of inflammatory factors, including leukotriene B4 (LTB4), prostaglandin E2 (PGE2) and thromboxane B2 (TXB2), were significantly decreased in hippocampus and cortex of APP/PS1 mice [45]. These findings suggested that DSS play a positive effective role in ameliorating oxidative stress and neuroinflammation and finally ameliorating cognition deficits in APP/PS1 mice [45], which are consistent with our predictions in this study.

However, several shortcomings should be recognized in the presented study. First, although we have integrated a large number of protein targets from published literatures, publicly available databases and network-based inference method, the incompleteness of current DTI networks may still exist. Second, as the intrinsic interactions and level of expression of multiple ingredients in the mixture of different plants are complicated, current study could not entirely reflect the actual functional actions of DSS in the human body. Finally, although recent experimental study had validated part of the predicted biological processes regulating by DSS, such as oxidative stress and inflammation [45], the remaining predicted targets and anti-AD mechanisms of DSS are necessary to be further validated by more comprehensive and in-depth wet-lab experiment in the future.

## Conclusion

The present study provides potential strategies for exploring the therapeutic mechanism of DSS against AD, which also indicates that systems pharmacology could be an effective infrastructure to decipher the compatibility and MOAs of the complex components in TCM prescription.

## Supplementary information

**Additional file 1: Table S1.** Detailed information of the 299 genes related with AD.

**Additional file 2: Table S2.** Detailed structural information of the 1,042 herbal ingredients in DSS.

**Additional file 3: Table S3.** The top 20 putative targets for each compound in DSS with known targets.

**Additional file 4: Table S4.** The 79 common targets exist in all the six herbs of DSS.

**Additional file 5: Table S5.** Detail information for the drug-target network of DSS.

**Additional file 6: Table S6.** Detailed information of GO terms enrichment analysis data of targets with degree equal or larger than 40.

**Additional file 7: Table S7.** Detailed information of KEGG pathway enrichment analysis data of targets with degree equal or larger than 40.

**Additional file 8: Table S8.** Detailed information of the integrated pathway on DSS for treatment of AD.

## Data Availability

The data is available and will be provided upon request to the corresponding author.

## Abbreviations

AD: Alzheimer’s disease
TCM: Traditional Chinese medicine
DSS: Danggui-Shaoyao-san decoction
DTIs: Drug–target interactions
HIA: Human intestinal absorption
BBB: Blood-brain barrier
DMTs: Disease-modifying treatments
BS: *Paeoniae Radix Alba*, BaiShao
DG: *Angelica Sinensis Radix*, DangGui
BZ: *Atractylodis Macrocephalae Rhizoma*, BaiZhu
CX: *Chuanxiong Rhizoma*, ChuanXiong
ZX: *Alismatis Rhizoma*, ZeXie
FL: *Poria*, FuLing
RCTs: Randomized controlled trials
MMSE: Mini-Mental State Examination
ADL: Activities of daily living
rCBF: Regional cerebral blood flow
MOAs: Mechanism of actions
HPLC: High-performance liquid chromatography
CTD: Comparative Toxicogenomics Database
HGMD: Human Gene Mutation Database
HIT: Herbal Ingredients Target Database
TCMID: Traditional Chinese Medicine Integrated Database
bSDTNBI: A balanced substructure-drug-target network-based inference
KR: Klekota-Roth
T-F: Target-function
GO: Gene ontology
PPAR: Peroxisome proliferator-activated receptor
ROS: Reactive oxygen species
BP: Biological process
MF: Molecular function
HIF-1: Hypoxia-inducible factor 1
IL-6: Interleukin-6
IL-1β: Interleukin-1β
MAPK: Mitogen-activated protein kinase
NF-*κ*B: Nuclear factor-kappa B
LPS: Lipopolysaccharide
BDNF: Brain-derived neurotrophic factor
NT3: Neurotrophin 3
Ach: Acetylcholine
TMP: Tetramethylpyrazine
MDA: Malondialdehyde
SOD: Superoxide dismutase
GSH: Glutathione
LTB4: Leukotriene B4
PGE2: Prostaglandin E2
TXB2: Thromboxane B2
